# Sn(IV)-Porphyrin-Based Nanostructures Featuring Pd(II)-Mediated Supramolecular Arrays and Their Photocatalytic Degradation of Acid Orange 7 Dye

**DOI:** 10.3390/ijms232213702

**Published:** 2022-11-08

**Authors:** Nirmal Kumar Shee, Hee-Joon Kim

**Affiliations:** Department of Chemistry and Bioscience, Kumoh National Institute of Technology, Gumi 39177, Korea

**Keywords:** Sn(IV)-porphyrin, coordination, supramolecular array, nanostructures, photocatalytic degradation of dyes

## Abstract

Two robust Sn(IV)-porphyrin-based supramolecular arrays (**1** and **2**) were synthesized via the reaction of *trans*-Pd(PhCN)_2_Cl_2_ with two precursor building blocks (**SnP^1^** and **SnP^2^**). The structural patterns in these architectures vary from 2D to 3D depending on the axial ligation of Sn(IV)-porphyrin units. A discrete 2D tetrameric supramolecule (**1**) was constructed by coordination of {(*trans*-dihydroxo)[5,10-bis(4-pyridyl)-15,20-bis(phenyl) porphyrinato]}tin(IV) (**SnP^1^**) with *trans*-PdCl_2_ units. In contrast, the coordination between the {(*trans*-diisonicotinato)[5,10-bis(4-pyridyl)-15,20-bis(phenyl)porphyrinato]}tin(IV) (**SnP^2^**) and *trans*-PdCl_2_ units formed a divergent 3D array (**2**). Axial ligation of the Sn(IV)-porphyrin building blocks not only alters the supramolecular arrays but also significantly modifies the nanostructures, including porosity, surface area, stability, and morphology. These structural changes consequently affected the photocatalytic degradation efficiency under visible-light irradiation towards acid orange 7 (AO) dye in an aqueous solution. The degradation efficiency of the AO dye in the aqueous solution was observed to be between 86% to 91% within 90 min by these photocatalysts.

## 1. Introduction

Industrial and agricultural activities emit hazardous chemicals into bodies of water threatening aquatic life and polluting drinking water [1,2,3]. Massive quantities of toxic chemicals are discharged into aquatic systems annually causing serious environmental problems. Azo dyes, the most common synthetic dye, account for more than 50% of global dye production because of their extensive use in the dyeing and textile industries. These dyes are stable under light and resistant to chemical oxidation; hence, they are not efficiently removed during conventional wastewater treatment. Azo dyes in bodies of water are fatal for aquatic life and have been a serious worldwide threat. Numerous methods have been developed to remove these toxic pollutants from wastewater [4,5,6,7]. Some notable physicochemical methods are adsorption [8], chemical precipitation [9], filtration [10], and advanced oxidation processes (AOPs) [11]. The AOP is the most promising method because it is simple to operate, less costly, has high degradation efficiency, and can decompose pollutants into CO_2_ and H_2_O. Pollutants are efficiently degraded by reactive oxygen species (ROS) generated in situ by an appropriate photocatalyst that absorbs light [12,13,14,15]. Photocatalysts that can efficiently use sunlight are constantly being sought because metal oxide-based photocatalysts lack visible light activity.

Free-base porphyrins and metalloporphyrins, types of porphyrinoids, are the most attractive building blocks of photofunctional materials that absorb visible light. Porphyrinoid-based nano- or micro-structured materials have received tremendous attention over the last two decades [16,17,18,19,20]. Their well-ordered and versatile geometrical features make them suitable not only for photocatalytic degradation but also in other fields: catalysis [21], solar energy conversion and storage [22], molecular recognition [23], and biomedical purposes [24]. Free-base porphyrins and metalloporphyrins self-assemble through various intermolecular interactions: hydrogen bonding, π–π stacking, van der Waals forces, metal–ligand coordination, and electrostatic interactions. Hydrophobic and hydrophilic effects further contribute to the self-organization of porphyrinoids in solution [25,26,27,28]. There are various methods to synthesize self-organized porphyrin nanomaterials: surfactant [29] and ionic self-assembly assisted [30], sonication [31], metal coordination [32], and reprecipitation [33]. Highly directional metal–ligand coordination has attracted particular attention because of its elaborate design leading to a variety of 2D or 3D architectures: ladders [34], rectangles [35], cube cages [36], wheel-type nanoring [37], coordination polymers [38], double-decker complexes [39], and molecular squares [40].

Pyridylporphyrins play a special role in supramolecular coordination assembly based on several attractive features [41,42]: (a) rigid planar geometries with interesting optical and redox properties; (b) conjugation with metal ions (Pd^2+^, Pt^2+^, Ru^2+^, Rh^2+^, and Re^+^) to form various coordination complexes used in catalysis, molecular recognition, and optoelectronics [43,44,45,46,47]. Sn(IV)-porphyrin complexes have been extensively used in the fabrication of functional nanomaterials: nanotubes [48], nanofibers [49], nanorods [50], nanocomposites [51], and nanosheets [52]. Sn(IV)-porphyrin centers readily adopt a hexacoordination geometry with two *trans*-oxyanion ligands (alkoxides or carboxylates) because of the oxophilic nature of Sn(IV) centers. The ditopic character of Sn(IV)-porphyrins is due to different functional groups in their axial and peripheral positions. Therefore, Sn(IV)-porphyrin-based building blocks are used for the fabrication of functional multiporphyrin supramolecular arrays and nanostructures [53,54,55,56,57,58]. Their attractive photophysical properties have been used in optoelectronic supramolecular systems [59,60].

We have explored Sn(IV)-porphyrin-based nanostructures using supramolecular assemblies. Complementary Zn(II)-Sn(IV)-Zn(II) porphyrin triads [17,18,32,50], the coordination of Sn(IV)-porphyrin with Ag(I) ions [15], and the ionic assembly of cationic Sn(IV)porphyrin [19] were studied for fabricating nanostructures, morphology control, and photocatalytic performance. We have further explored nanomaterial-based supramolecular assemblies featuring well-defined dimensions and innate porosity, such as porphyrin squares, for photocatalytic degradation of pollutant dyes. The design and selection of suitable building blocks with well-defined shapes and dimensions are challenging tasks for the development of specific nanostructures with efficient performance.

We here report two porphyrin-based supramolecular architectures (2D and 3D) synthesized by coordination between the peripheral pyridyl groups of Sn(IV)-porphyrin and Pd(II) complexes. Furthermore, we intensively investigated the nanostructures for the photocatalytic degradation of pollutant dyes in aqueous solutions. Acid Orange 7 (AO) dye was selected as the target contaminant in this study because AO is chemically stable, nonbiodegradable, mutagenic, and potentially carcinogenic.

## 2. Results and Discussion

### 2.1. Synthesis and Spectroscopic Characterization

**Scheme 1:**
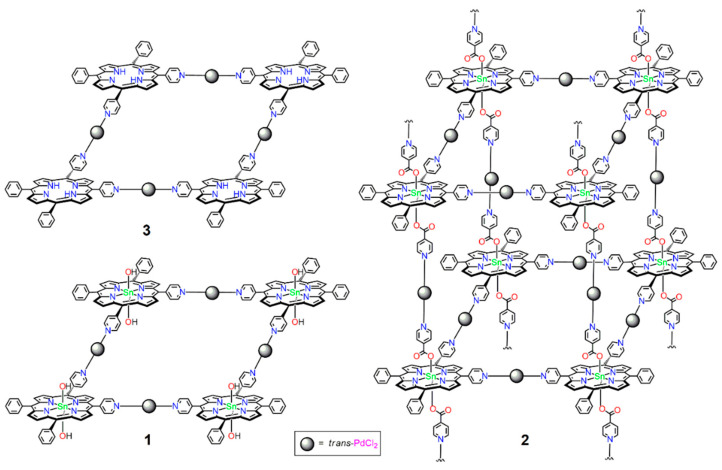
Chemical structures of representative supramolecular arrays.

**Scheme 2:**
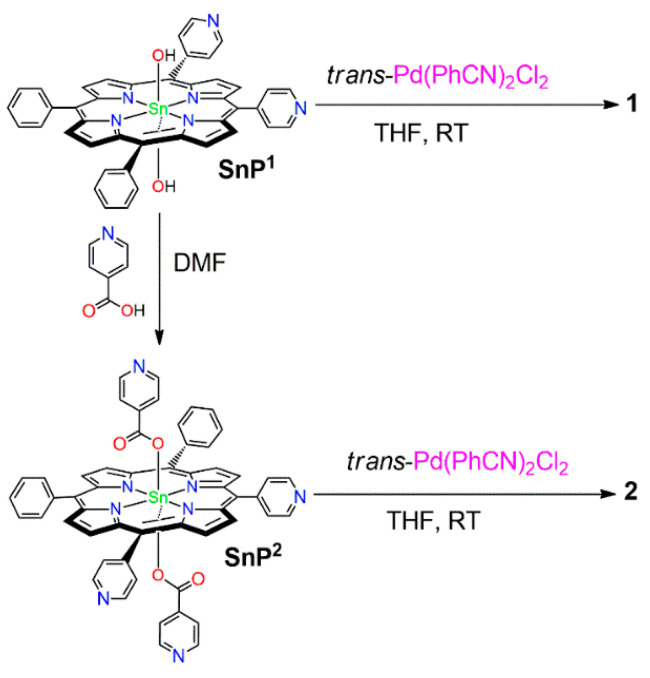
The syntheses of **1** and **2**.

Two supramolecular multiporphyrin architectures were designed as illustrated in Scheme 1: a discrete 2D tetrameric supramolecule **1** and a divergent 3D supramolecular array **2**. **1** was synthesized by using **SnP^1^** and *trans*-bis(benzonitrile)dichloropalladium(II) [*trans*-Pd(PhCN)_2_Cl_2_] as the building block and linkage source, respectively, according to the reported procedure by Drain et al. [61,62]. **SnP^1^** has two pyridyl donors at 90°, and *trans*-Pd(PhCN)_2_Cl_2_ serves as a two-site acceptor at 180° generated by the dissociation of labile benzonitrile ligands (Scheme 2). The reaction of **SnP^1^** with *trans*-Pd(PhCN)_2_Cl_2_ in THF at room temperature produced **1**. We later modified **1** with two axial isonicotinato-ligands to synthesize **2**. A divergent 3D supramolecular array of **2** can be developed when modified **1** is further reacted with *trans*-Pd(PhCN)_2_Cl_2_. However, isonicotinic acid reacts with Pd(II) and Sn(IV) centers of **1** and disrupts the closed 2D tetrameric structure of **1**. As an alternative strategy for the construction of a 3D supramolecular array, **SnP^2^** was used as a monomeric building block because it provides four pyridyl donors that are directly three-dimensional (Scheme 2). Although Pd(II) centers can randomly link pyridyl donors between **SnP^2^** complexes, it should be noted that **SnP^2^** is assembled into divergent 3D arrays through the reaction with *trans*-Pd(PhCN)_2_Cl_2_. The structure of **2** depicted in Scheme 1 is a representative 3D array. For comparative studies, we prepared free-base porphyrin-based closed 2D tetrameric array **3** using 5,10-bis(4-pyridyl)-15,20-bis(phenyl)porphyrin. All synthesized compounds were fully characterized by various spectroscopic techniques: elemental analysis, ^1^H NMR, FTIR, ESI–MS, UV–vis spectroscopy, fluorescence spectroscopy, PXRD, and FESEM.

The ^1^H NMR spectra of **SnP^1^**, **SnP^2^**, **1**, and **2** are shown in the Supplementary Materials (Figures S1–S4). Figure S3 shows a more complex ^1^H NMR spectrum of **1** than that of **SnP^1^** with a downfield shift of pyridyl protons which indicates the Pd(II)–pyridine complexation (Figure S2) [63]. The NMR spectrum of **SnP^1^** exhibits a pair of doublets ascribed to both α-pyridyl (H_2,6_) protons at 9.09 ppm and β-pyridyl (H_3,5_) protons at 8.31 ppm. (H_2,6_) protons significantly shift downfield to 9.57 ppm after Pd(II) binds with pyridyl, while the resonance shift and the splitting of β-pyridyl (H_3,5_) protons of **1** are less affected (8.47 ppm). The resonance of both phenyl and β-pyrrole protons split of **1** (due to the difference in the outer and inner square environments) to exhibit a smaller downfield shift as compared to **SnP^1^**. Similarly, α-pyridyl (H_2,6_) protons of porphyrin present in **SnP^2^** appear as a doublet at 9.09 ppm. After coordination with Pd(II), these protons exhibit significant downfield shifts and appear at 9.56 ppm in **2**. The α-pyridyl protons of the axial isonicotinato ligand of **2** also shift downfield (7.62 ppm to 7.93 ppm) after coordination with Pd(II). The peaks of phenyl and β-pyrrole protons of **2** shift slightly downfield compared to those of **SnP^2^**.

The ESI–MS spectra of **SnP^1^**, **SnP^2^**, and **1** are shown in Figures S5–S7. The molecular ion peaks of [**SnP^1^** + H]^+^^1^ and [**SnP^2^** + H]^+^^1^ appear at 769.09 and 979.23, respectively (Figures S5 and S6). As shown in Figure S7, the base peak of [**1** − 4Cl]^+4^ appears at 909.01. Other small fragments ([**1** − 2Cl]^+2^ at 1853.52, [**1** − 3Cl]^+3^ at 1224.01, and [**1** − 5Cl]^+5^ at 720.21) with low intensity are also observed. The appearance of the base peak and fragmentation of **1** in ESI–MS support the existence of a supramolecular array as illustrated in Scheme 1. The FTIR spectra of **1** and **2** along with their starting porphyrins (**SnP^1^** and **SnP^2^**) are shown in Figure S8. The absence of a characteristic band of nitrile stretching (*ca.* 2220 cm^−1^) in **1** and **2** indicates that pyridyl *N* atoms coordinate with Pd(II) by completely replacing PhCN during the reaction of *trans*-Pd(PhCN)_2_Cl_2_ with either **SnP^1^** or **SnP^2^**. Additionally, the characteristic band of carboxylate stretching of **SnP^2^** and **2** appears at 1660 cm^−1^ (Figure S8).

**Figure 1:**
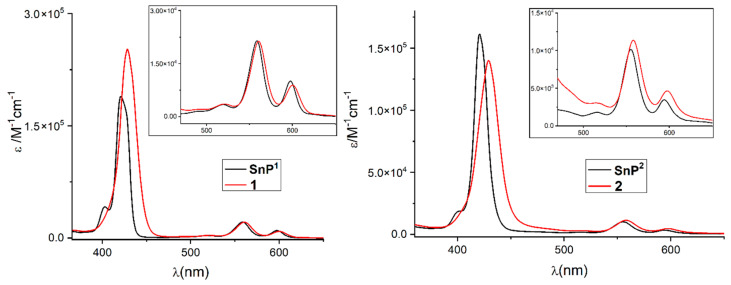
The UV–vis absorption spectra of **SnP^1^**, **SnP^2^**, **1**, and **2** in chloroform.

The optical properties of **SnP^1^**, **SnP^2^**, **1**, and **2** were investigated using UV–vis spectroscopy in chloroform (Figure 1). The Soret band of **SnP^1^** displays sharp peaks at 420 nm along with Q-bands at 519, 558, and 598 nm. These bands are redshifted by **1** and the Soret band appears at 428 nm upon complexation with Pd(II). A shift of 8 nm confirms the coordination of pyridyl *N* to the Pd(II) complex in **1**. Q-bands also redshift and appear at 521, 560, and 601 nm. In contrast, the Soret band of **SnP^2^** appears at 420 nm, and Q-bands appear at 516, 555, and 594 nm. All these bands of **2** are redshifted compared to bands of **SnP^2^**. Array **2** exhibits a Soret band at 429 nm, while Q-bands appear at 517, 558, and 598 nm. Therefore, bathochromic shifts and broadening confirm the formation of Pd(II)-*N* bonds. These results are consistent with those previously reported [62,63,64,65].

**Figure 2:**
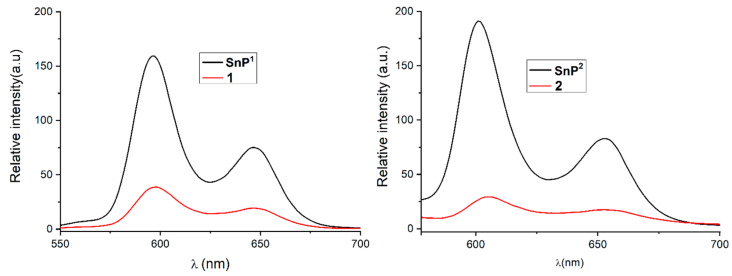
The fluorescence spectra of **SnP^1^**, **SnP^2^**, **1**, and **2** in chloroform.

The fluorescence spectra of **SnP^1^**, **SnP^2^**, **1**, and **2** in chloroform are shown in Figure 2. The fluorescence spectrum of **SnP^1^** shows two emission bands at 596 and 646 nm, which are redshifted to 598 and 648 nm, respectively, with a lower relative intensity of **2** due to complexation with Pd(II). Similarly, **SnP^2^** displays a two-band pattern at 601 and 653 nm (Figure 2). These bands redshift to 605 and 654 nm after forming a complex with Pd(II) in **2**. In all cases, the emission intensity is largely quenched (up to 90%) upon ligation and changes the peak-to-peak ratio. This is due to the energy transfer between porphyrin rings via π−π stacking and the heavy metal ion effect, which accelerates the singlet-to-triplet excited-state intersystem crossing [62].

The thermal stabilities of **1** and **2** were measured using thermogravimetric analysis (TGA) under He flow (Figure S9). A slight weight loss is observed in the case of **1** up to 300 °C owing to the loss of volatile molecules. A major weight loss occurs in the range of 300–400 °C because of the destruction of the framework (loss of the Pd(II) linkage with porphyrin). Similarly, the major weight loss of **2** occurs in the range of 350–450 °C due to the decomposition of the framework. These results indicate that **1** and **2** are stable up to 300–350 °C. We also examined the crystallinity of these arrays by analyzing their PXRD patterns (Figure S10) and confirmed them as amorphous and less crystalline as observed in Figure S10. N_2_ sorption isotherms were recorded at 77 K in a liquid nitrogen bath to determine the permanent porosities of **1**, **2**, and **3** (Figure S11). The estimated Brunauer–Emmett–Teller (BET) surface areas are 47, 82, and 32 m^2^ g^−1^ for **1**, **2**, and **3**, respectively. The presence of permanent porosity within these supramolecular arrays can enable access to metalloporphyrin sites in the pores of small molecules, thus providing higher catalytic performance toward dyes.

**Figure 3:**
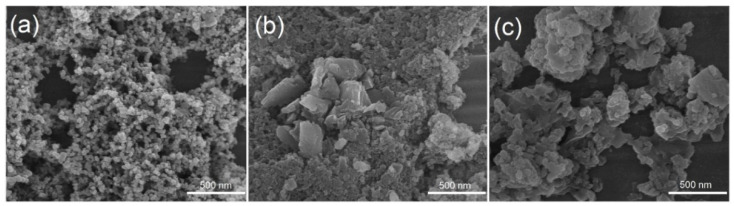
The FE-SEM images of **1** (**a**), **2** (**b**), and **3** (**c**).

The morphologies of **1** and **2** were observed using field-emission scanning electron microscopy (FESEM) (Figure 3). We compared the surface morphology of **1** with those of **3** to determine the effect of the metal on porphyrin. Sponge-like nanoparticles are observed in **1** with average lengths ranging from 25 to 65 nm as confirmed by DLS studies (Figure S12). The particles are larger in **3** and interconnected compared to those in **1**. These observations indicate that the presence of the (*trans*-dihydroxo)-Sn(IV) center in the porphyrin unit significantly affects the morphology of **1** compared to that of **3**. The average particle size with a high population of **2** ranges from 25 to 100 nm, while particles with lower populations have sizes within 325–375 nm. The surface morphology of **SnP^1^** and **SnP^2^** is not sufficient to describe the specific shapes (Figure S13).

### 2.2. Photocatalytic Performance for the Degradation of AO

**Figure 4:**
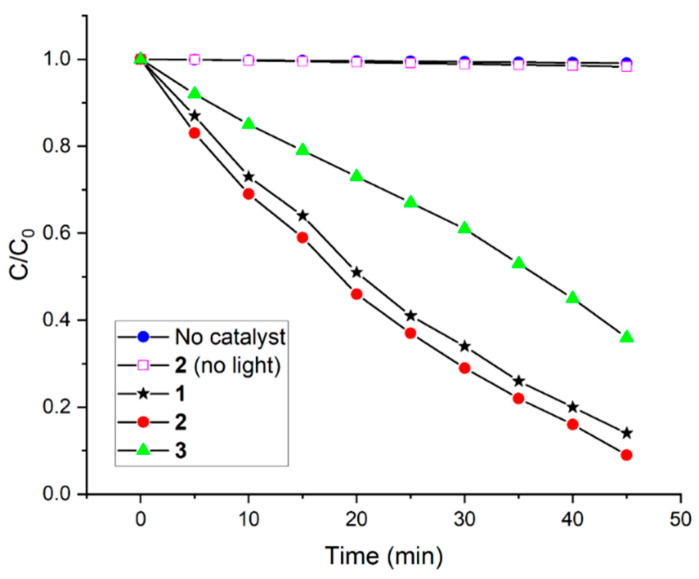
The photocatalytic degradation of AO dye in an aqueous solution (pH 7, T = 298 K) under visible-light irradiation in the presence of photocatalysts **1**, **2**, and **3**.

The photocatalytic degradation activity of **1**, **2**, and **3** were investigated by degrading AO in an aqueous solution under visible-light irradiation. As shown in Figure S14, approximately 30 min is required to reach the adsorption–desorption equilibrium; 11%, 16%, and 9% of AO are adsorbed by **1**, **2**, and **3**, respectively. This presumably indicates that these porphyrin arrays possess suitable surface areas and porosity for AO adsorption. The time-dependent absorption spectra of AO in the presence of **2** under visible-light irradiation are shown in Figure S15. A negligible AO decay is observed in the absence of any photocatalyst or visible light (Figure 4).

Photocatalysts and visible light are necessary for AO degradation. Figure S15 shows that the absorbance at 485 nm decreases with increasing visible light irradiation time indicating the photochemical degradation of AO in the presence of the photocatalyst. All photocatalysts (**1**, **2**, and **3**) show significant AO degradation (Figure 4). AO degradation in the presence of the photocatalyst can be described by its degradation efficiency expressed (*C*_0_ − *C*)/*C*_0_, where *C*_0_ is the initial concentration of AO dye, and *C* is the concentration at time *t*. The observed AO degradation efficiency values of **1**, **2**, and **3** are 86%, 91%, and 64%, respectively (Figure 4). **2** shows better performance in AO degradation than either **1** or **3**. We applied a pseudo-first-order kinetic rate equation ln(*C*_0_/*C*) = *kt* to further interpret the reaction kinetics for AO decay. This equation is used when the *C*_0_ of the dye is low, and *k* is the pseudo-first-order degradation rate constant. The reaction kinetics of AO degradation is obtained based on the data plotted in the graph in Figure 4 as shown in Figure S16. The first-order rate constants of AO degradation are 0.043 min^−1^, 0.047 min^−1^, and 0.021 min^−1^ by **1**, **2**, and **3**, respectively (Figure S16). These results are notable compared to the other reported values of AO degradation (Table 1).

The reusability of **1**, **2**, and **3** is necessary for practical applications and was determined by recycling tests of **2** by degrading AO under visible-light irradiation (Figure S17). **2** maintained 96% of its initial degradation performance after 10 consecutive cycles indicating its stability. The morphologies of **1** and **2** were also examined after degrading AO to confirm their stability. PXRD patterns (Figure S18) and FESEM images (Figure S19) of reused **1** and **2** are similar to their unused images indicating that the framework of these photocatalysts is adequately stable after the photocatalytic reaction.

We attempted to optimize the reaction conditions in terms of temperature, the pH of the AO solution, and the AO dye/catalyst ratio. Degradation experiments were performed at different temperatures to estimate the effect of temperature on AO degradation by **2**. The degradation efficiency increases with an increase in temperature (Figure S20). We prepared aqueous solutions of AO at different pH values (2–12) to determine the effect of the pH of the dye solution on degradation. The pH of the aqueous AO solution affects the AO degradation rate (Figure S21). Figure S21 shows that the rate of degradation increases from pH 2 to 7 followed by a decrease to pH 12. A strong acidic or basic medium likely disintegrates the framework of **2**. The degradation rate is affected more in a basic medium than in an acidic medium. The effect of the dye/catalyst ratio on AO degradation was examined using various concentrations of AO solution (5, 10, 15, 20, 30, 35, and 40 mg L^−1^) and a constant amount of **2** (10 mg). Degradation rates decrease with an increase in the AO concentration (Figure S22).

We have proposed a possible mechanism for the photodegradation of pollutant dyes using porphyrin-based nanomaterials in aqueous solutions [15,32]. The mechanism comprises five steps: (a) the photocatalyst absorbs light under visible-light irradiation and AO from an aqueous solution. Valence band (VB) electrons are promoted to the conduction band (CB) after crossing the bandgap forming electron–hole (*e*^−^/*h*^+^) pairs at the surface of the photocatalyst. Strong intermolecular π–π interaction among porphyrin molecules intensifies the electronic delocalization within the photocatalysts, which can be minimized by the recombination energy of excited electrons; (b) photogenerated holes (*h*^+^) react with H_2_O to generate a highly reactive hydroxyl radical (^•^OH); (c) excited electrons react with dissolved O_2_ molecules to produce reactive superoxide radical anions (O_2_^−•^), (d) these photogenerated superoxide radical anions and hydroxyl radicals react with the AO dye; (e) the dye is degraded into smaller molecules (CO_2_ and H_2_O). The mechanism consisting of five steps for porphyrin-based frameworks (**P**) is shown in Equations (1)–(5):

**P** + *hν* → P* (*e*^−^ + *h*^+^) (1)

H_2_O + *h*^+^ → ^•^OH + H^+^ (2)

O_2_ + 2*e*^−^ → O_2_^−•^ (3)

^•^OH + AO → degraded products (4)

O_2_^−•^ + AO → degraded products (5)

Radical trapping experiments were performed to detect the photogenerated reactive species during the photocatalytic AO degradation by **2** [19,50]. We used *tert*-butanol (^t^BuOH) to capture hydroxyl radicals (^•^OH) and *p*-benzoquinone (*p*-BQ) as superoxide radical anions (O_2_^−•^) during the photodegradation in the presence of **2** (Figure S23). The results reveal that the degradation rate is severely affected by ^t^BuOH and *p*-BQ. Superoxide radical anions (O_2_^−•^) are the major reactive species compared to hydroxyl radicals (^•^OH) for catalytic AO degradation. We observed that **SnP^1^** and **SnP^2^** show very low efficiency to degrade AO (Figure S23). The photocatalytic activity of **2** was investigated under various monochromatic light wavelengths (Figure S24). The variation of the wavelength-dependent AO photodegradation shows that optical absorption significantly contributes toward solar energy conversion and photocatalytic performance. In addition, **2** shows a small degradation ability even at λ > 700 nm.

**Figure 5:**
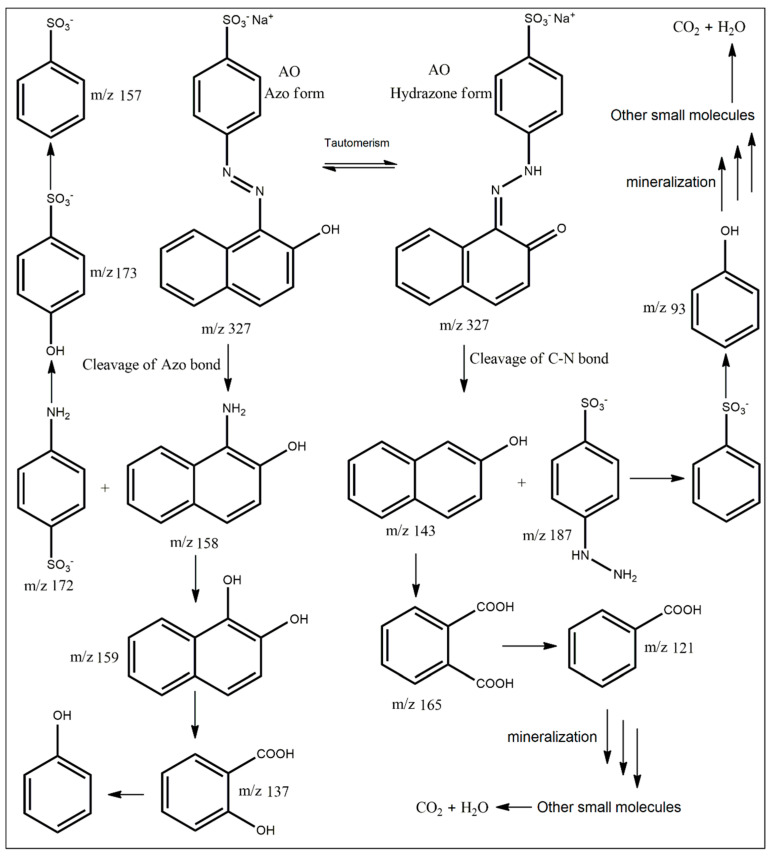
Possible intermediates formed in the AO degradation by **2**.

We investigated the degradation products of AO by **2** after irradiating under visible light. The reaction mixture obtained after 30 min was analyzed using ESI–MS (Figure S25). New peaks are observed in mass spectra confirming the degradation of AO into smaller molecules [69]. For an AO sample before the photodegradation experiment, few fragments of AO occurred in the ESI-MS measurement. Figure 5 and Table S1 show the possible intermediates of AO degradation. The base peak (*m*/*z* = 327; [AO − Na]^+^) corresponds to anionic AO and exists in the solution as keto–enol tautomers. AO can be fragmented in two ways: (a) the cleavage of the azo bond by the hydroxyl radical (^•^OH) and the formation of two low molecular weight fragments: 1-amino-2-naphthol (*m*/*z* 158) and sulfanilic acid (*m*/*z* 172). The primary amine (*m*/*z* 172) may hydrolyze to generate either *m*/*z* 173 or *m*/*z* 157. However, the successive oxidation of other amines forms 1,2-dihydroxy naphthalene (*m*/*z* 159), followed by salicylic acid (*m*/*z* 137) to phenol (*m*/*z* 93); (b) The cleavage of the C–N bond forms lower molecular fragments with *m*/*z* 143 and 187. The successive ring-opening oxidation of 2-naphthol forms *ortho*-phthalic acid (*m*/*z* 165), followed by benzoic acid (*m*/*z* 121). However, the successive oxidation and hydrolysis of the fragment (*m*/*z* 187) form benzene sulfonic acid (*m*/*z* 157), followed by phenol (*m*/*z* 93). Finally, all lower molecular weight intermediates are further fragmented and mineralized into H_2_O and CO_2_. The total organic carbon (TOC) value was calculated to quantify the AO removal by photocatalysts [66]. TOC removal percentages by **1**, **2**, and **3** were 69%, 75%, and 59%, respectively.

## 3. Materials and Methods

All chemicals were purchased from Sigma–Aldrich (Seoul, Korea) and used without further purification unless stated otherwise. 5,15-bis(4-pyridyl)-10,20-bis(phenyl)porphyrin (**H_2_P**) [61], *trans*-Pd(PhCN)_2_Cl_2_ [62], and array **3** [63] were synthesized according to the previously reported procedures. THF was purified by distillation over sodium/benzophenone ketyl solution while distilled pyrrole was obtained using calcium hydride. ^1^H NMR spectra were obtained using a Bruker BIOSPIN/AVANCE III 400 spectrometer at 293 K (Bruker BioSpin GmbH, Silberstreifen, Rheinstetten, Germany). Steady-state UV–vis and fluorescence spectra were recorded using a Shimadzu UV-3600 spectrophotometer and a Shimadzu RF-5301PC fluorescence spectrophotometer, respectively (Shimadzu, Tokyo, Japan). The FTIR spectra were obtained using a Shimadzu FTIR-8400S spectrophotometer (Shimadzu, Tokyo, Japan). Electrospray ionization mass (ESI-MS) spectra were recorded using a Thermo Finnigan linear ion trap quadrupole mass spectrometer (Thermo Fisher Scientific, Waltham, MA, USA). Elemental analysis was performed using an EA 1110 Fisons analyzer (Used Lab Machines Limited, London, UK). Field emission scanning electron microscopy (FESEM) images were obtained using a MAIA III (TESCAN, Brno, Czech Republic). Powder X-ray diffraction (PXRD) patterns were obtained using a Bruker AXS D8 Advance powder X-ray diffractometer (Bruker; Billerica, MA, USA). Thermogravimetric analysis (TGA) was performed using an Auto-TGA Q500 instrument (TA Instruments, New Castle, DE, USA). Dynamic light scattering (DLS) experiments were performed using a NanoBrook 90Plus DLS size analyzer (Brookhaven, NY, USA). The Brunauer–Emmett–Teller (BET) surface area was determined with an analyzer (BELSORP-mini volumetric adsorption equipment) using N_2_ adsorption isotherms at 77 K.

### 3.1. Material Synthesis

#### 3.1.1. {(*trans*-Dihydroxo)[5,10-bis(4-Pyridyl)-15,20-bis(phenyl)porphyrinato]}tin(IV) **SnP^1^**

**H_2_P** (0.099 g, 0.16 mmol) was dissolved in pyridine (20 mL). SnCl_2_∙2H_2_O (0.22 g, 0.95 mmol) was added to the solution and refluxed for 12 h. Pyridine was removed under vacuum, and the residue was dissolved in CHCl_3_. The mixture was filtered through a celite pad, and the solvent in the filtrate was evaporated. The obtained solid was dissolved in 40 mL of a mixed solvent (10 mL H_2_O + 30 mL THF). K_2_CO_3_ (0.325 g, 1.45 mmol) was added to the above reaction mixture and refluxed for 12 h. THF was removed at reduced pressure, and the mixture was allowed to precipitate at 0 °C. The solid precipitate was filtered and dried in an oven. Recrystallization of the precipitate using CHCl_3_/CH_3_CN formed a reddish solid **SnP^1^**. Yield: 90 mg (73%). Anal. Calcd for C_42_H_28_N_6_O_2_Sn: C, 65.73; H, 3.68; N, 10.95; R, 19.64. Found: C, 65.35; H, 3.94; N, 10.50; R, 20.23. ^1^H NMR (400 MHz, CDCl_3_, ppm): *δ* −7.50 (s, 2H, Sn-OH), 7.78–7.84 (m, 8H, *m*,*p*-phenyl), 8.27–8.31 (m, 8H, *o*-phenyl + 3,5-pyridyl), 9.09–9.19 (m, 12H, β-pyrrole + 2,6-pyridyl). FTIR (KBr pellet, ν/cm^−1^): 1590 (s), 1465 (w), 1400 (w), 1340 (w), 1260 (w), 1200 (w), 1070 (s), 1020 (vs), 790 (vs), 700 (w), 660 (w), 550 (br). UV–vis (CHCl_3_, nm): *λ*_max_(log *ε*); 403(4.6), 420(5.27), 519(3.5), 558(4.42), 598(4.03). Emission (CHCl_3_, nm): *λ*_max_ 596, 646.

#### 3.1.2. {(*trans*-Diisonicotinato)[5,10-bis(4-pyridyl)-15,20-bis(phenyl)porphyrinato]}tin(iv) **SnP^2^**

A mixture of **SnP^1^** (50 mg, 0.065 mmol) and isonicotinic acid (20 mg, 0.16 mmol) in DMF (20 mL) was stirred at room temperature. After 24 h, the solvent was removed *under a vacuum*. The product was purified by column chromatography (SiO_2_, eluent: CH_2_Cl_2_/MeOH = 98:2) and recrystallized from CHCl_3_/*n*-hexane to obtain violet microcrystals of **SnP^2^** (50 mg, 79%). Anal. Calcd. for C_54_H_34_N_8_O_4_Sn: C, 66.34; H, 3.51; N, 11.46; R, 18.69. Found: C, 66.01; H, 3.82; N, 11. 38; R, 18.79. ^1^H NMR (400 MHz, CDCl_3_): *δ* 4.79 (d, *J* = 6 Hz, 4H, isonicotinato-H_α_), 7.62 (d, *J* = 6 Hz, 4H, isonicotinato-H_β_), 7.80–7.85 (m, 6H, *m*,*p*-phenyl), 8.20–8.23 (m, 8H, *o*-phenyl + 3,5-pyridyl), 9.09 (d, *J* = 5.6 Hz, 4H, 2,6-pyridyl), 9.19–9.26 (m, 8H, β-pyrrole). FTIR (KBr pellet, ν/cm^−1^): 1656 (s), 1590 (s), 1540 (w), 1405 (w), 1310 (br), 1210 (w), 1140 (w), 1060 (w), 1025 (s), 790 (s), 760 (s), 670 (br). UV–vis (CHCl_3_, nm): *λ*_max_(log *ε*); 402(4.29), 420(5.21), 516(3.25), 555(4.01), 594(3.56). Emission (CHCl_3_, nm): *λ*_max_ 601 and 653.

#### 3.1.3. Supramolecular Array **1**

*trans*-Pd(PhCN)_2_Cl_2_ (0.019 g, 0.05 mmol) was added with stirring to a solution of **SnP^1^** (0.038 g, 0.05 mmol) in 10 mL of THF. After 1 h, the brown precipitate was filtered, washed with 5 mL THF, and dried in air. Yield: 35 mg (74%). Anal Calcd. for C_168_H_112_Cl_8_N_24_O_8_Pd_4_Sn_4_: C, 65.03; H, 4.12; N, 10.57; R, 20.28. Found: C, 64.87; H, 4.34; N, 10.44; R, 20.35. ^1^H NMR (400 MHz, CDCl_3_): *δ* −7.36 (s, 2H, Sn-OH), 7.88 (m, 6H, *m*,*p*-phenyl), 8.35 (d, *J* = 6.4 Hz, 4H, *o*-phenyl), 8.47 (d, *J* = 6.4 Hz, 4H, 3,5-pyridyl), 9.21–9.29 (m, 8H, β-pyrrole), 9.57 (d, *J* = 5.6 Hz, 4H, 2,6-pyridyl). FTIR (KBr pellet, ν/cm^−1^): 1610 (s), 1420 (s), 1320 (br), 1225 (br), 1135 (w), 1030 (vs), 860 (w), 765 (br), 700 (vs). UV–vis (CHCl_3_, nm): *λ*_max_(log *ε*); 428(5.40), 521(3.55), 560(4.36), 601(3.98). Emission (CHCl_3_, nm): *λ*_max_ 598 nm and 648.

#### 3.1.4. Supramolecular Array **2**

*trans*-Pd(PhCN)_2_Cl_2_ (0.038 g, 0.1 mmol) was added with stirring to a solution of **SnP^2^** (0.049 g, 0.05 mmol) in 15 mL of THF. After 1 h, the red precipitate was filtered, washed with 5 mL of THF, and dried in air. Yield: 61 mg (89%). Anal Calcd. for {C_54_H_34_Cl_4_N_8_O_4_Pd_2_Sn}_n_: C, 63.87; H, 4.00; N, 10.41; R, 21.72. Found: C, 63.65; H, 4.29; N, 10.22; R, 21.84. ^1^H NMR (400 MHz, CDCl_3_): *δ* 4.88 (d, *J* = 6 Hz, 4H, isonicotinato-H_α_), 7.85–7.93 (m, 10H, isonicotinato-H_β_ + *m*,*p*-phenyl), 8.35 (d, *J* = 6.4 Hz, 4H, *o*-phenyl), 8.46 (d, *J* = 6.4 Hz, 4H, 3,5-pyridyl), 9.20–9.30 (m, 8H, β-pyrrole), 9.56 (d, *J* = 5.6 Hz, 4H, 2,6-pyridyl). FTIR (KBr pellet, ν/cm^−1^): 1660 (s), 1610 (s), 1475 (w), 1415 (w), 1210 (w), 1070 (w), 1020 (vs), 795 (vs), 700 (w), 545 (br). UV–vis (CHCl_3_, nm): *λ*_max_(log *ε*); 429(5.15), 517(3.48), 558(4.04), 598(3.72). Emission (CHCl_3_, nm): *λ*_max_ 605, 654.

### 3.2. Photocatalytic Degradation Investigation

The photocatalytic performance of porphyrin-based supramolecular arrays (**1**–**3**) was investigated by the photodegrading AO in an aqueous solution. AO was photodegraded under the irradiation of a 150 W xenon arc lamp with a UV cut-off filter (ABET Technologies, Old gate lane Milford, CT, USA) at 298 K. In a typical procedure, 10 mg of the photocatalyst was added to 200 mL of an aqueous solution of AO (15 mg L^−1^, distilled water with pH 7.0) with stirring. The reaction mixture was allowed to stand in the dark for 30 min to reach the adsorption–desorption equilibrium. After irradiation with visible light, a 3 mL suspension was collected at regular intervals. The photocatalyst was separated from the solution by centrifugation and collected by filtration through a membrane filter. The AO concentration was determined by measuring the absorbance at 485 nm using a UV–Vis spectrophotometer.

## 4. Conclusions

We synthesized two porphyrin-based supramolecular architectures (**1** and **2**) via the reaction of *trans*-Pd(PhCN)_2_Cl_2_ with two precursor building blocks (**SnP^1^** and **SnP^2^**). The structural patterns varied from 2D to 3D depending on the axial ligation of the starting Sn(IV)-porphyrin units. Discrete 2D tetrameric supramolecule **1** was constructed by the coordination of **SnP^1^** with *trans*-PdCl_2_ units. In contrast, the coordination between **SnP^2^** and *trans*-PdCl_2_ units formed a divergent 3D array **2**. Axial ligation of Sn(IV)-porphyrin building blocks not only alters the supramolecular arrays but also significantly modifies the nanostructures, including porosity, surface area, stability, and morphology. These structural changes consequently affected the photocatalytic degradation efficiency under visible light irradiation towards AO in an aqueous solution. The degradation efficiency of AO in an aqueous solution was observed to be between 86% to 91% within 90 min by these photocatalysts. The high dye degradation efficiency, low catalyst loading, and high reusability make these photocatalysts more promising than conventional photocatalysts, such as TiO_2_, ZnO, or other photocatalysts [66,67,68,69,70,71,72,73,74,75,76,77,78,79]. We believe that our current reports on photocatalysts hold great promise for the future treatment of dyeing wastewater.

## Figures and Tables

**Table 1:** A comparison of the photodegradation efficiency of AO with various photocatalysts.

Photocatalysts	Rate Constant (min^−1^)	Reference
TiO_2_ P-25/Cu	0.041	[66]
TiO_2_ P-25	0.045	[66]
MnFe_2_O_4_-γGO	0.019	[67]
ZnFe_2_O_4_-C_3_N_4_(2:3)	0.012	[68]
Fe^3+^-doped TiO_2_	0.011	[69]
Au-Fe(1:1)/TiO_2_	0.014	[70]
ZnS-Mn_3_O_4_ (1.5%wt)	0.054	[71]
2-line ferrihydrite	0.026	[72]
Co-Fe nitride	0.024	[73]
Fe^0^/H_2_O_2_	0.054	[74]
ZnO	0.021	[75]
ZnO-Ag	0.051	[75]
g-CN/TiO_2_	0.016	[76]
[Cu(HLsalpyca)(NO_3_)]_n_/H_2_O_2_	0.015	[77]
CNP/B-BiVO_4_/WO_3_	0.014	[78]
BiOBr/rGO/ZnPc(CO_2_H)_4_(0.25)	0.023	[79]
**1**	0.043	this study
**2**	0.047	this study
**3**	0.021	this study

## Data Availability

Not applicable.

## Supplementary Materials

The following supporting information can be downloaded at: https://www.mdpi.com/article/10.3390/ijms232213702/s1.
