# Supplementary material for: Sn(IV)-Porphyrin-Based Nanostructures Featuring Pd(II)-Mediated Supramolecular Arrays and Their Photocatalytic Degradation of Acid Orange 7 Dye
Source: Int J Mol Sci. 2022 Nov 8;23(22):13702. doi: 10.3390/ijms232213702 (PMC9696627; doi:10.3390/ijms232213702)
Supplement: Supplementary file 1 [file ijms-23-13702-s001.zip › ijms-2018715-supplementary.pdf]

Supplementary Materials

Sn(IV)-Porphyrin-Based Nanostructures Featuring Pd(II)-Mediated Supramolecular Arrays and Their Photocatalytic Degradation of Acid Orange 7 Dye

Nirmal Kumar Shee and Hee-Joon Kim*

Department of Chemistry and Bioscience, Kumoh National Institute of Technology
61 Daehak-ro, Gumi 39177, Republic of Korea

List of contents:

Figure S1. The ^{1}H NMR spectrum of {(*trans*-dihydroxo)[5,10-bis(4-pyridyl)-15,20-bis(phenyl)porphyrinato]}tin(IV) (**SnP**^{1}) in $CDCl_3$.

Figure S2. The ^{1}H NMR spectrum of {(*trans*-diisonicotinato)[5,10-bis(4-pyridyl)-15,20-bis(phenyl)porphyrinato]}tin(IV) (**SnP**^{2}) in $CDCl_3$.

Figure S3. The ^{1}H NMR spectrum of array **1** in $CDCl_3$.

Figure S4. The ^{1}H NMR spectrum of array **2** in $CDCl_3$, which was measured after the addition of 2 equiv. of *trans*-$Pd(PhCN)_2Cl_2$ into the **SnP**^{2} solution of 2.0×10^{-6} M.

Figure S5. The electrospray ionization mass (ESI-MS) spectrum of **SnP**^{1}.

Figure S6. The ESI-MS spectrum of **SnP**^{2}.

Figure S7. The ESI-MS spectrum of **1**.

Figure S8. The FTIR spectra of **1** and **2** along with **SnP**^{1} and **SnP**^{1}.

Figure S9. The thermogravimetric analysis (TGA) curves of **1** and **2**.

Figure S10. The powder X-ray diffraction (PXRD) patterns of **1** and **2**.

Figure S11. The adsorption and desorption isotherms of N_2 for **1**, **2**, and **3** at 77 K.

Figure S12. Particle size determination by dynamic light scattering (DLS) for **1** and **2** in THF.

Figure S13. The field emission scanning electron microscopy (FE-SEM) images of **SnP**^{1} (a) and **SnP**^{2} (b).

Figure S14. The adsorption of AO on **1**, **2**, and **3**.

Figure S15. The time-dependent absorption spectra of AO in the presence of **2** under visible-light irradiation.

Figure S16. The kinetics of the photocatalytic degradation of AO under visible-light irradiation by photocatalysts **1**, **2**, and **3**.

Figure S17. The typical catalytic cycles (up to 10 consecutive cycles) of photocatalyst **2** for the degradation of AO.

Figure S18. The powder X-ray diffraction (PXRD) patterns of **1** and **2** after use for photocatalytic degradation of AO.

Figure S19. The typical FE-SEM image of photocatalyst **1** and **2** after the degradation of AO.

Figure S20. The effect of temperature on the degradation of AO by **2**.

Figure S21. The effect of pH of the AO solution for photodegradation by **2**.

Figure S22. The effect of the AO concentration on the photodegradation by **2** (10 mg).

Figure S23. The effect of various scavengers for the degradation of AO in the presence of **2** under visible-light irradiation ([*p*-BQ]$_0$ = [^{t}BuOH]$_0$ = 5 mM, pH 7.0, T = 298 K). **SnP^1** and **SnP^2** were used as catalysts for comparison.

Figure S24. The photocatalytic activities of **2** at different wavelengths for the degradation of AO.

Figure S25. The ESI-MS spectrum (negative ion mode) of the reaction mixture of AO in the presence of **2** after 30 min of visible-light irradiation.

Table S1. Degraded products from the photodegradation of AO identified by ESI-MS.

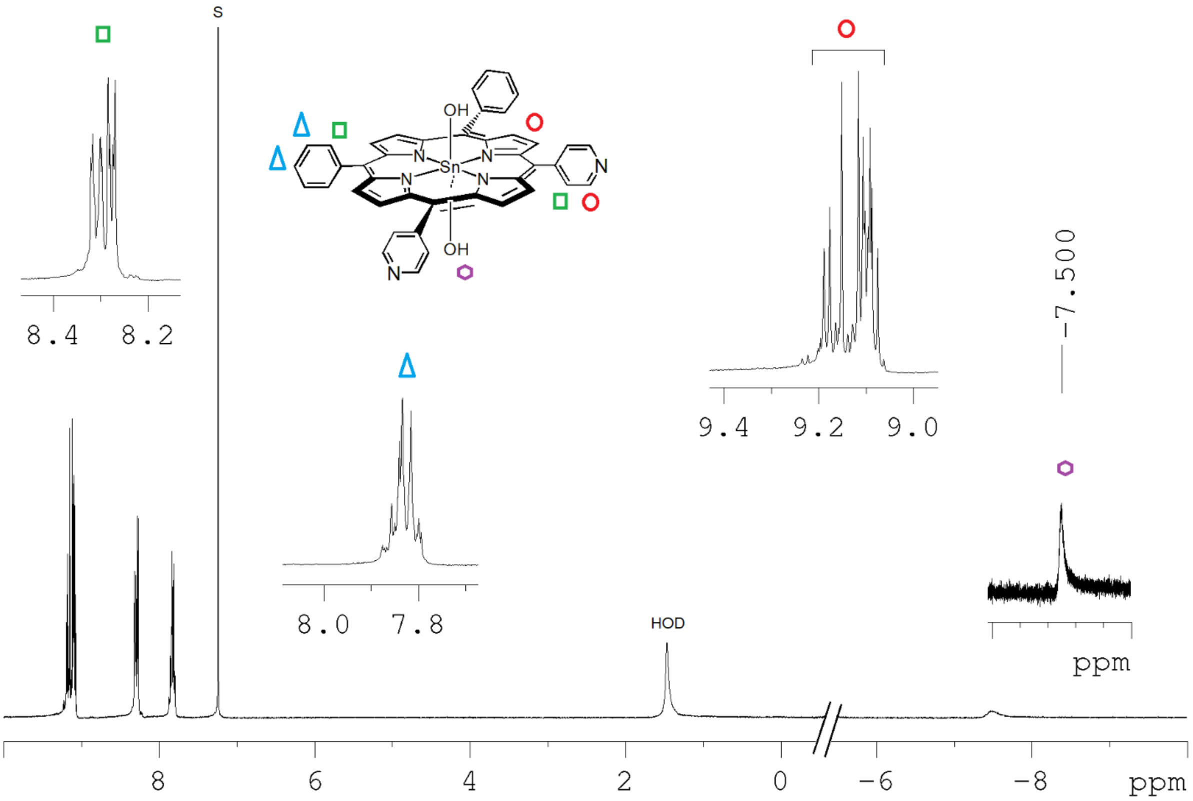

Figure S1. The ^{1}H NMR spectrum of {(*trans*-dihydroxo)[5,10-bis(4-pyridyl)-15,20-bis(phenyl)porphyrinato]}tin(IV) (**SnP¹**) in $CDCl_3$.

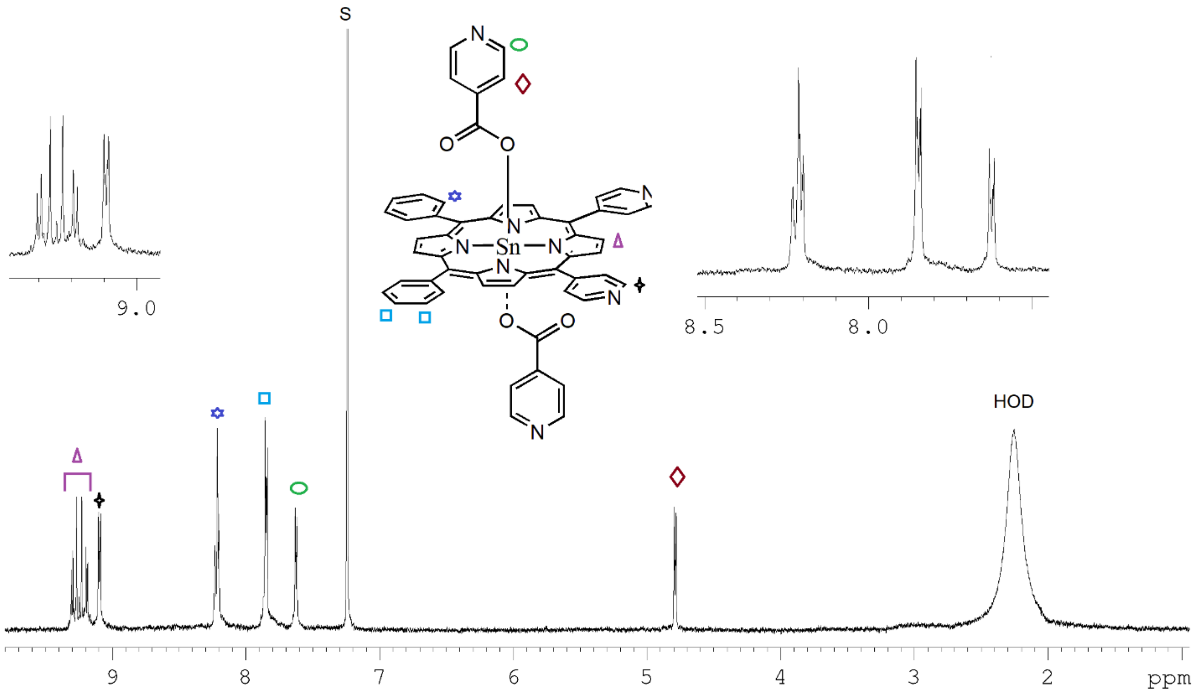

Figure S2. The ^{1}H NMR spectrum of {(*trans*-diisonicotinato)[5,10-bis(4-pyridyl)-15,20-bis(phenyl)porphyrinato]}tin(IV) (**SnP^{2}**) in $CDCl_3$.

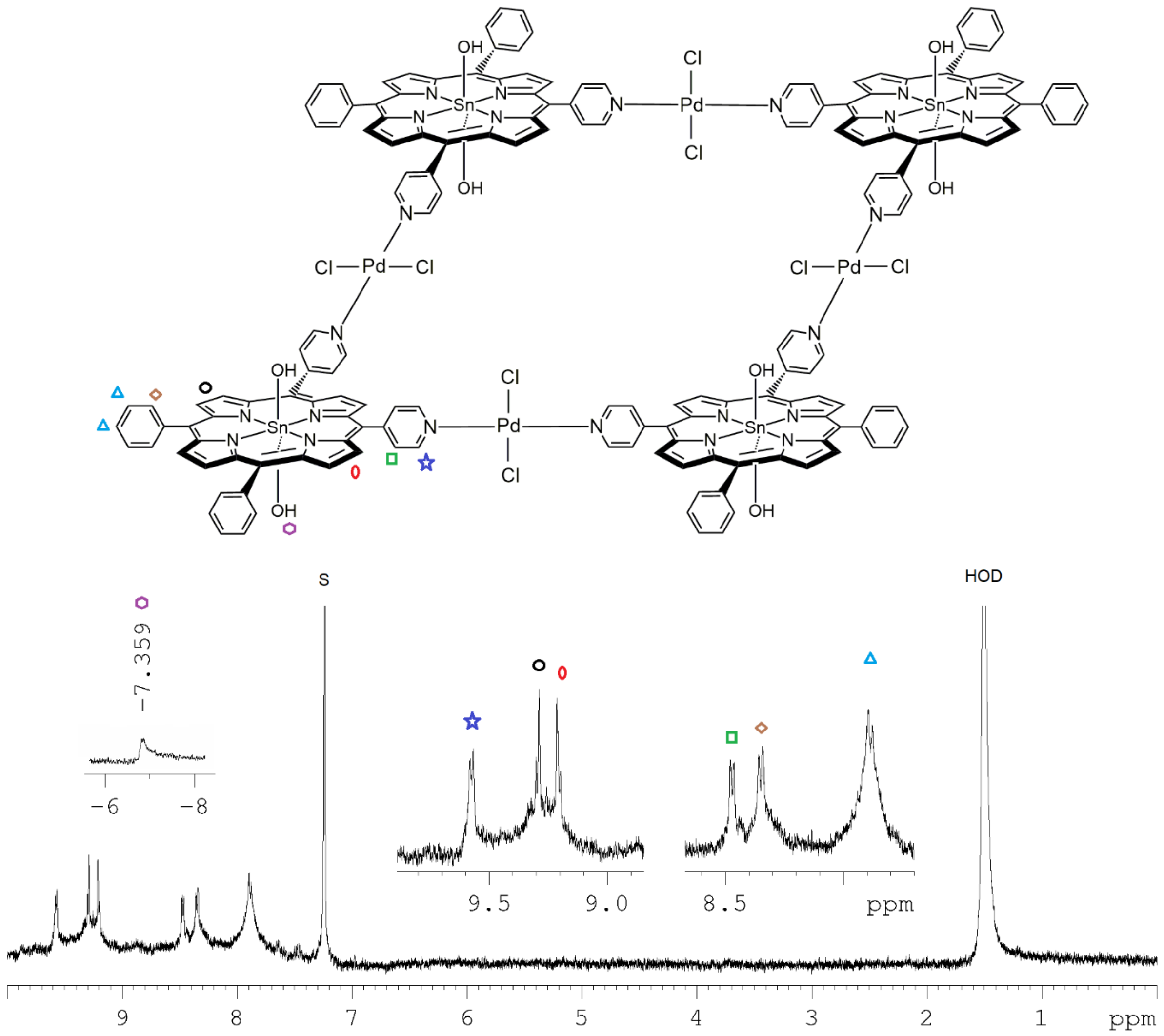

Figure S3. The ^{1}H NMR spectrum of array **1** in $CDCl_3$.

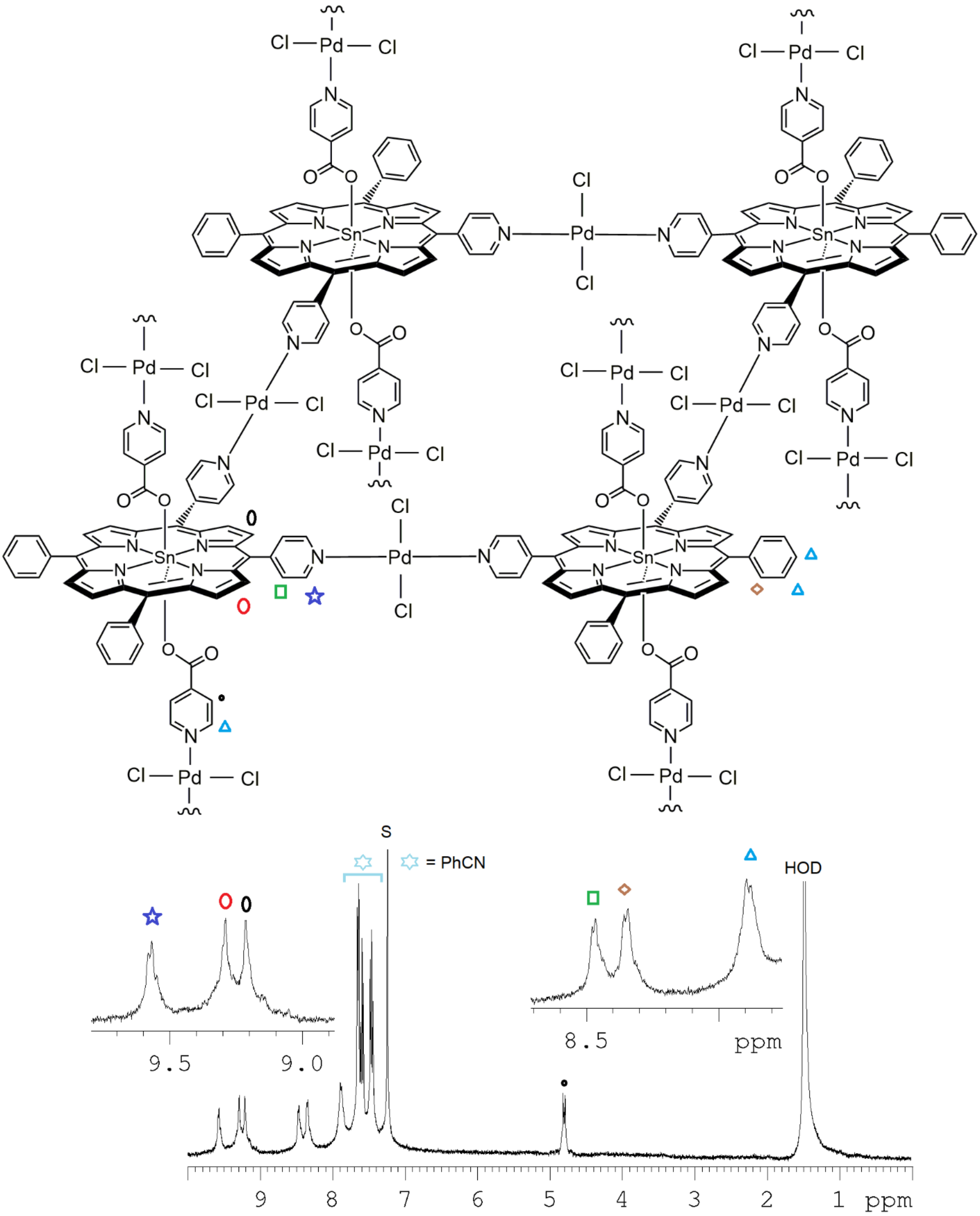

Figure S4. The ^{1}H NMR spectrum of array **2** in $CDCl_3$, which was measured after the addition of 2 equiv. of *trans*-$Pd(PhCN)_2Cl_2$ into the **SnP²** solution of 2.0×10^{-6} M.

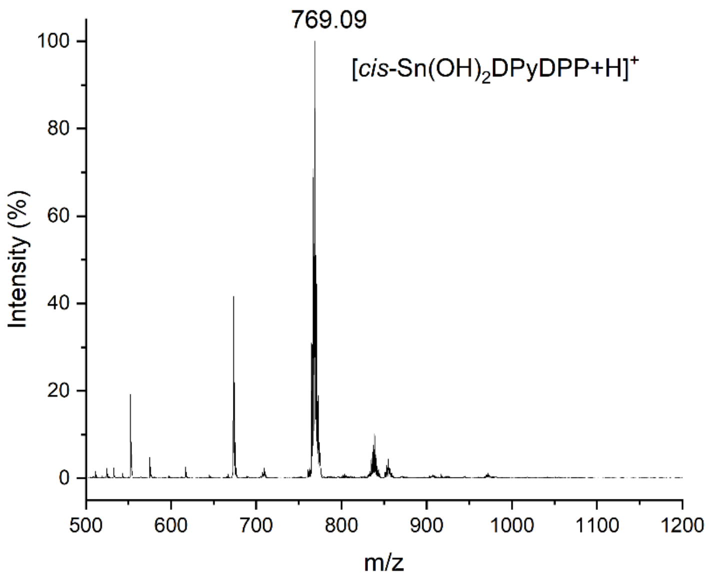

Figure S5. The electrospray ionization mass (ESI-MS) spectrum of **SnP1**.

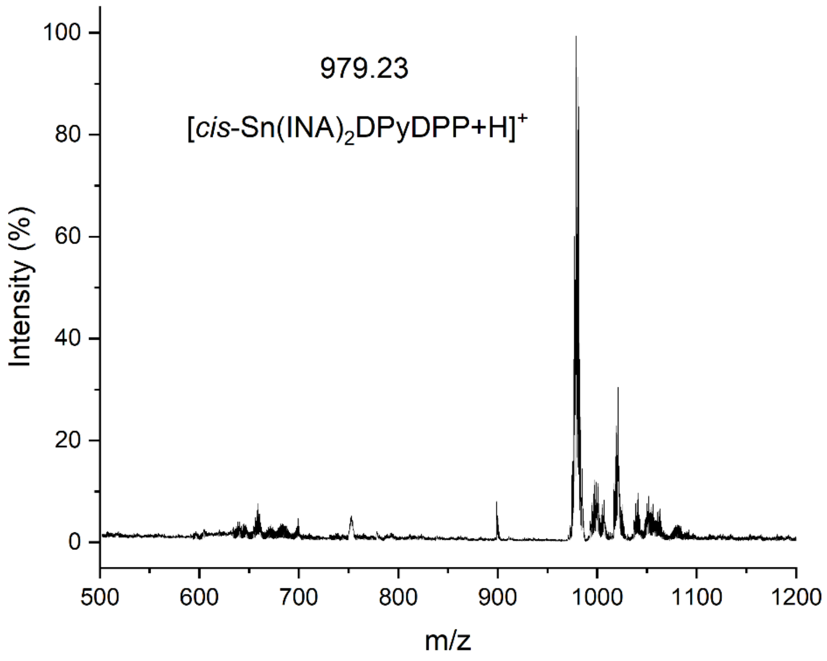

Figure S6. The ESI–MS spectrum of **SnP2**.

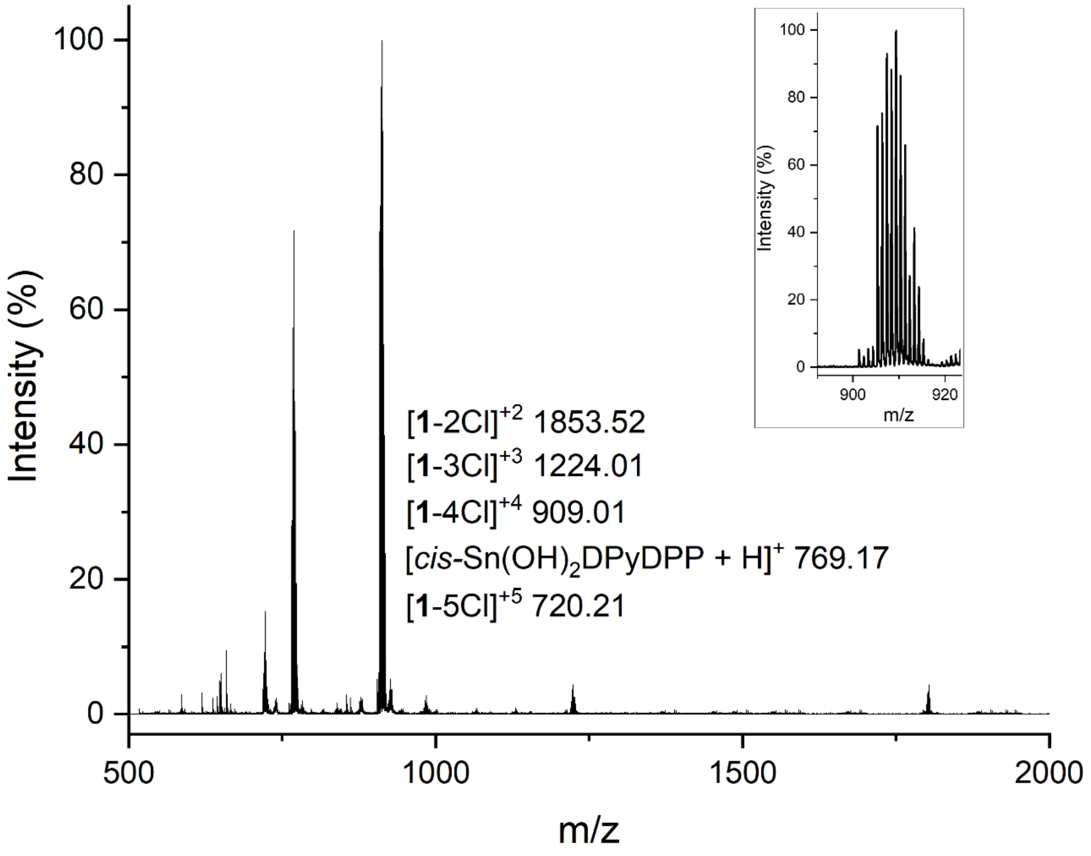

Figure S7. The ESI–MS spectrum of **1**.

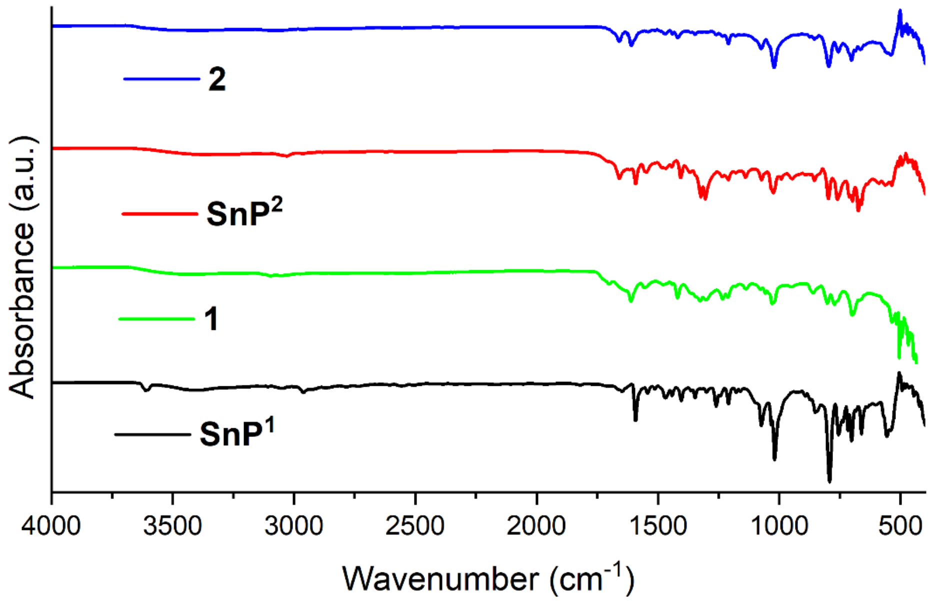

Figure S8. The FT-IR spectra of **1** and **2** along with **SnP**[1] and **SnP**[1].

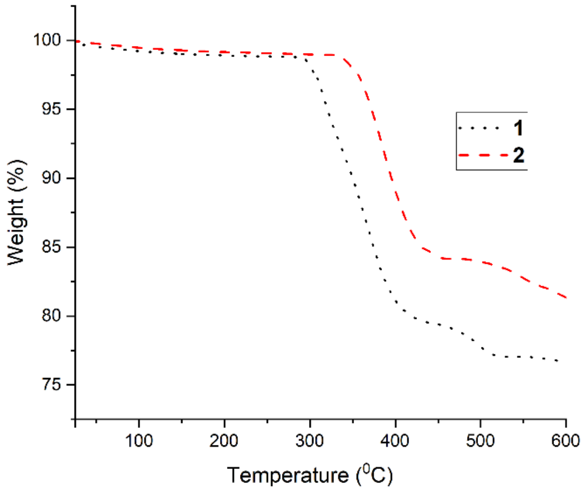

Figure S9. The thermogravimetric analysis (TGA) curves of **1** and **2**.

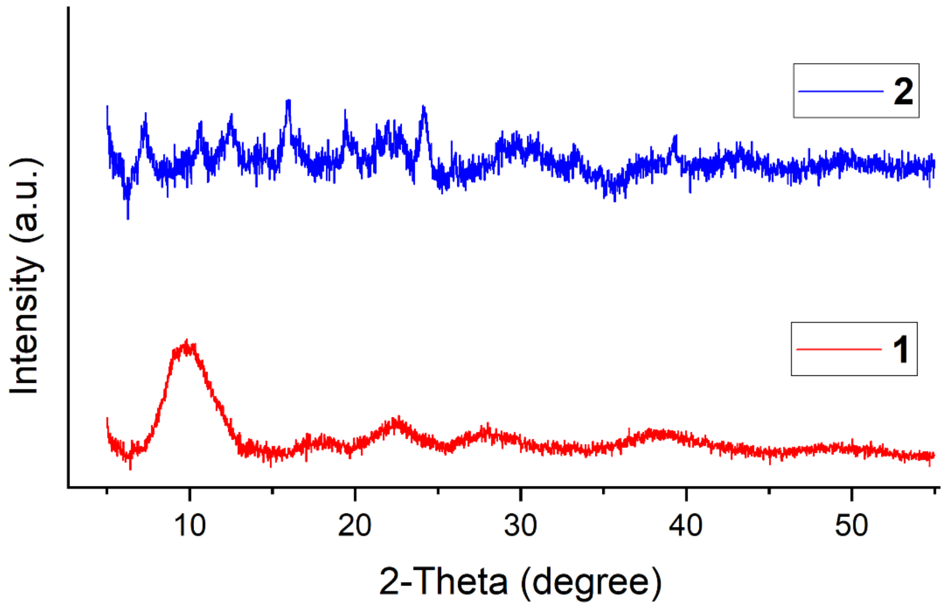

Figure S10. The powder X-ray diffraction (PXRD) patterns of **1** and **2**.

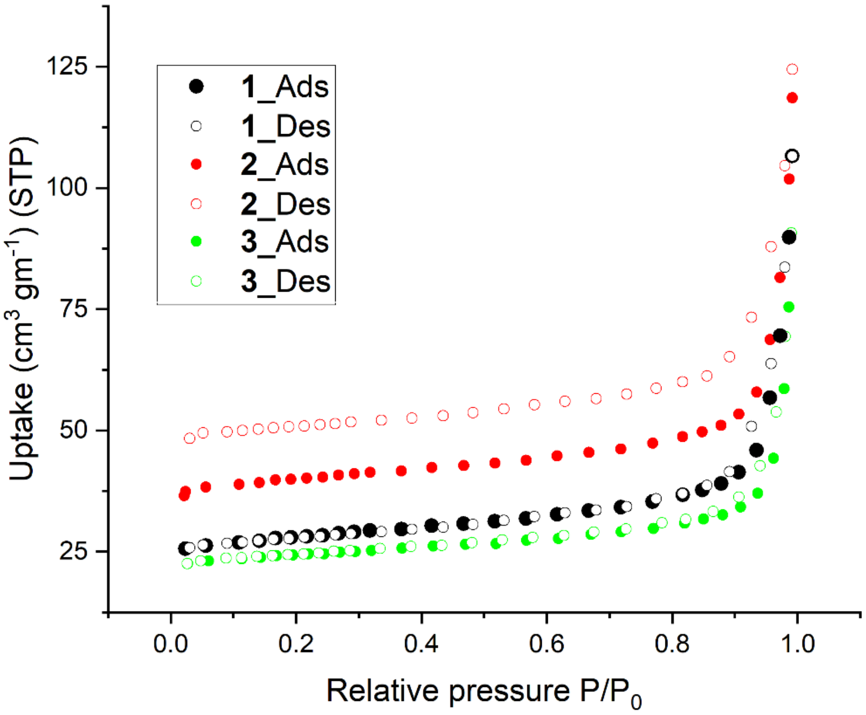

Figure S11. The adsorption and desorption isotherms of N_2 for **1**, **2**, and **3** at 77 K.

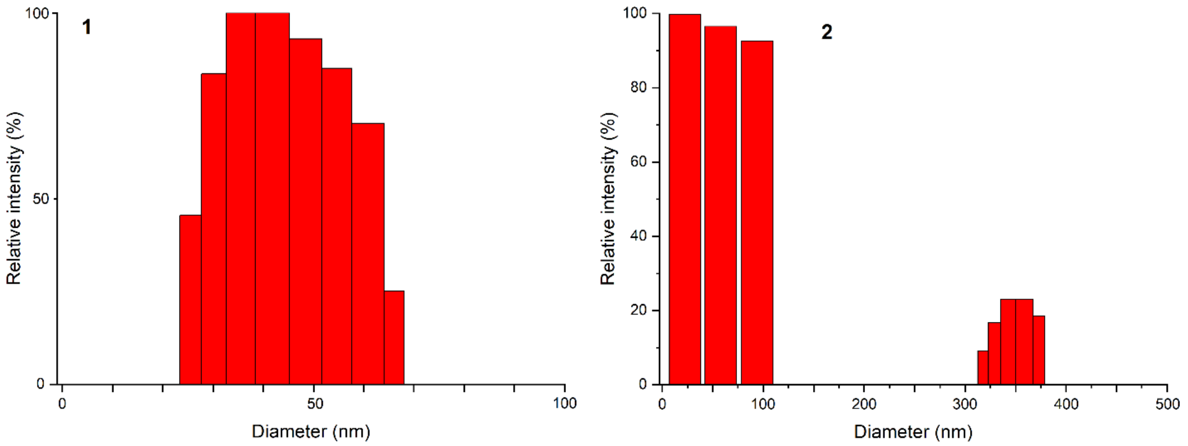

Figure S12. The particle size determination by dynamic light scattering (DLS) of **1** and **2** in THF.

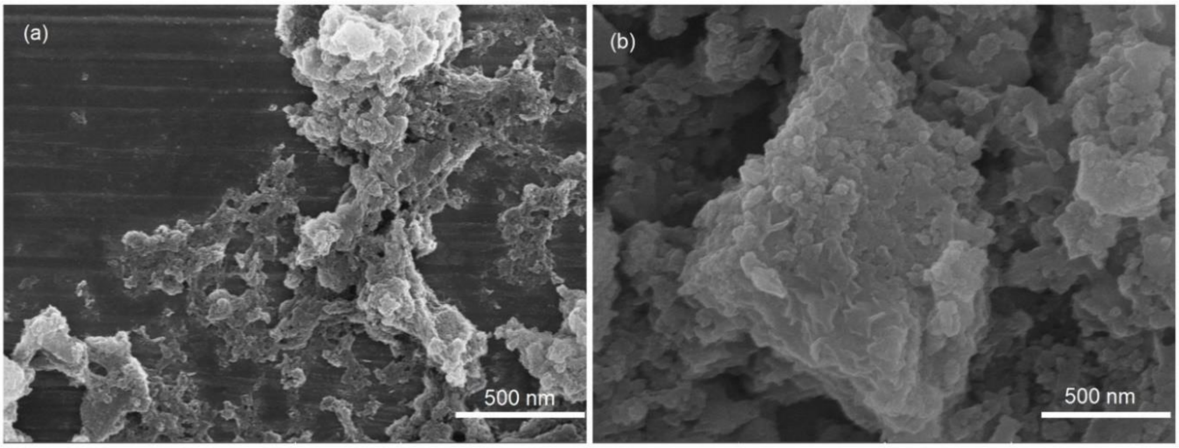

Figure S13. The field emission scanning electron microscopy (FESEM) images of **SnP[1]** (a) and **SnP[2]** (b).

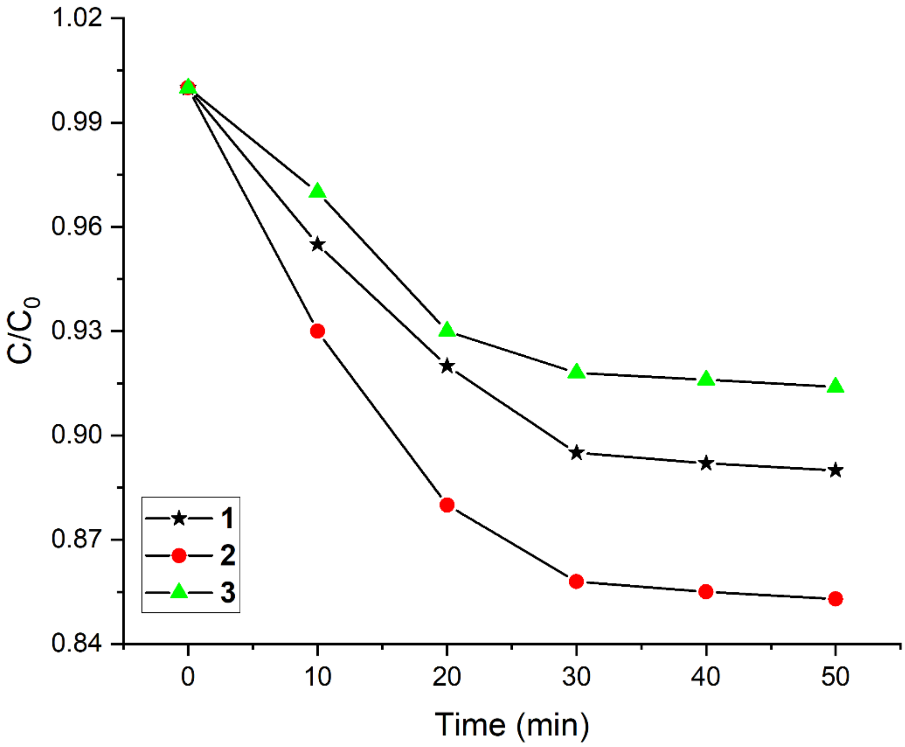

Figure S14. The adsorption of AO on **1**, **2**, and **3**.

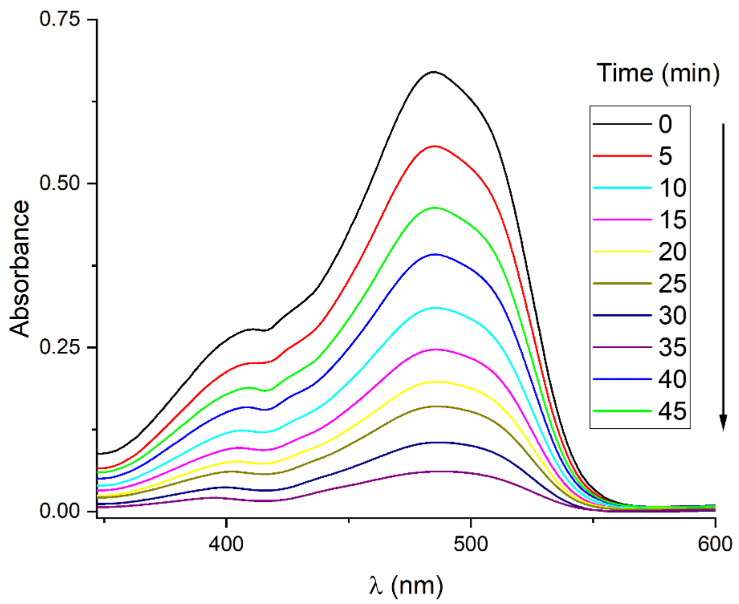

Figure S15. The time-dependent absorption spectra of AO in the presence of **2** under visible-light irradiation.

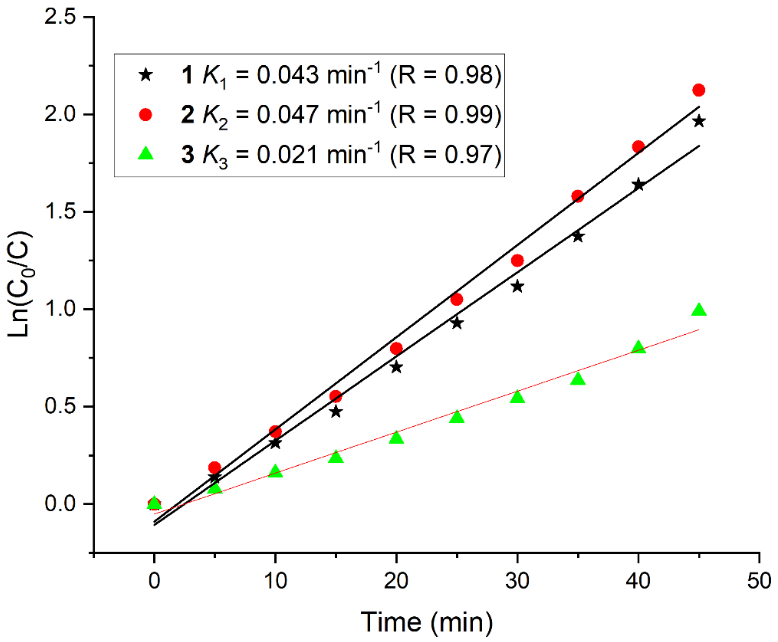

Figure S16. The kinetics of the photocatalytic degradation of AO under visible-light irradiation by **1**, **2**, and **3**.

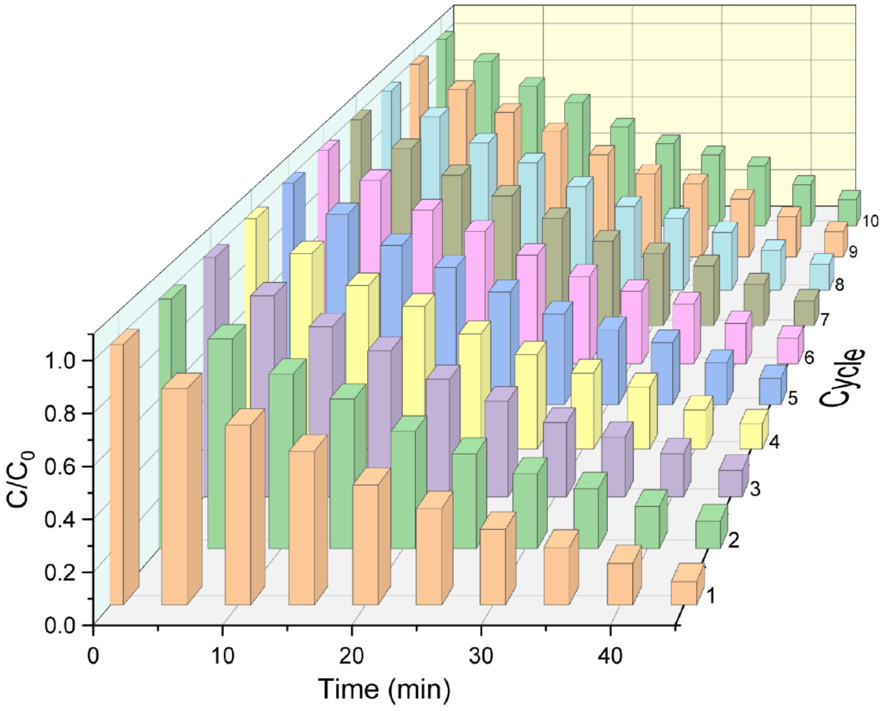

Figure S17. The typical catalytic cycles (up to 10 consecutive cycles) of **2** for the degradation of AO.

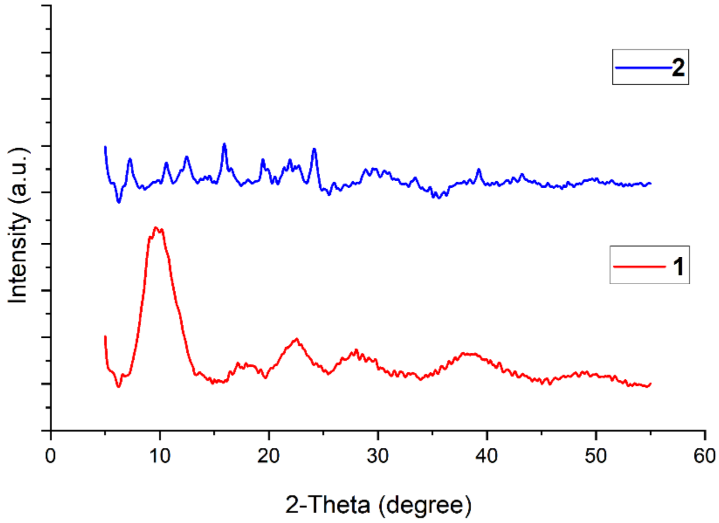

Figure S18. The PXRD patterns of **1** and **2** after use for photocatalytic degradation of AO.

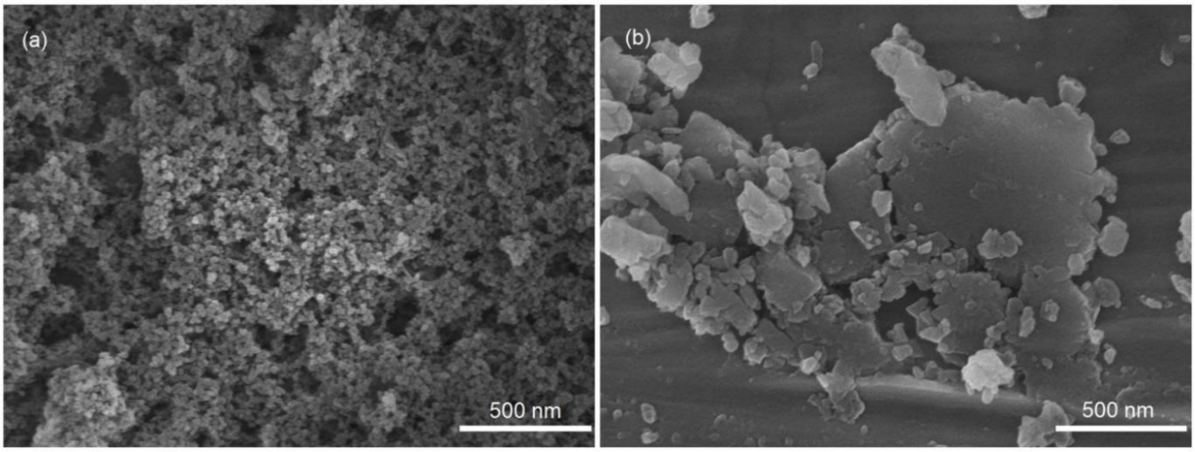

Figure S19. The FESEM image of photocatalyst **1** and **2** after AO degradation.

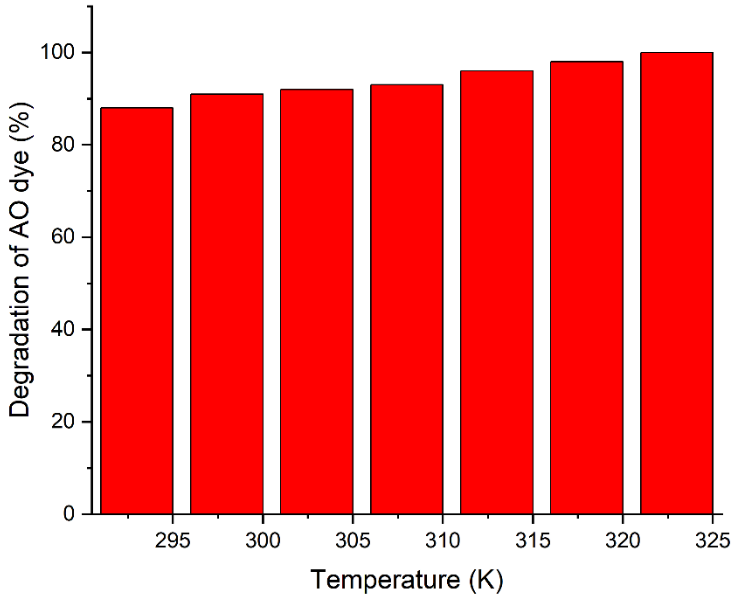

Figure S20. The effect of temperature on AO degradation by **2**.

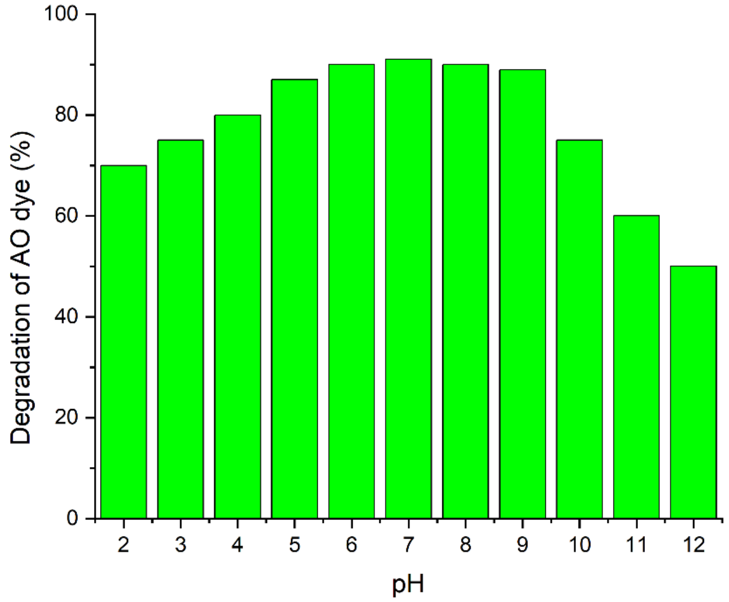

Figure S21. The effect of pH of the AO solution on photodegradation by **2**.

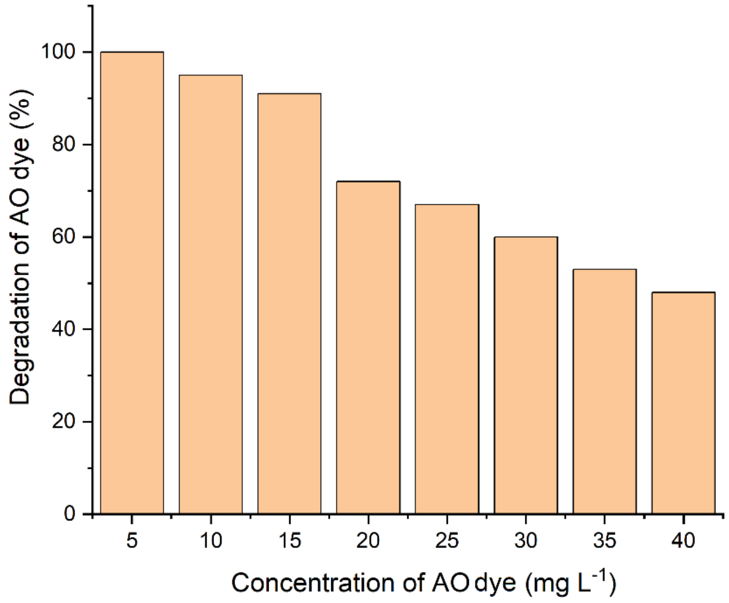

Figure S22. The effect of the AO concentration on photodegradation by **2** (10 mg).

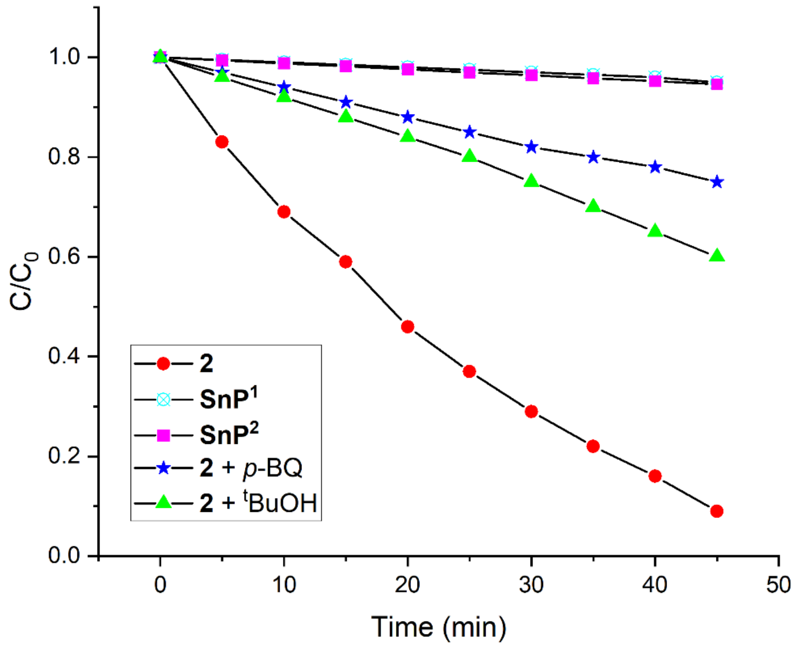

Figure S23. The effect of various scavengers for AO degradation by **2** under visible-light irradiation ($[p\text{-BQ}]_0 = [^t\text{BuOH}]_0 = 5$ mM, pH 7.0, T = 298 K). **SnP^1** and **SnP^2** were used as catalysts for comparison.

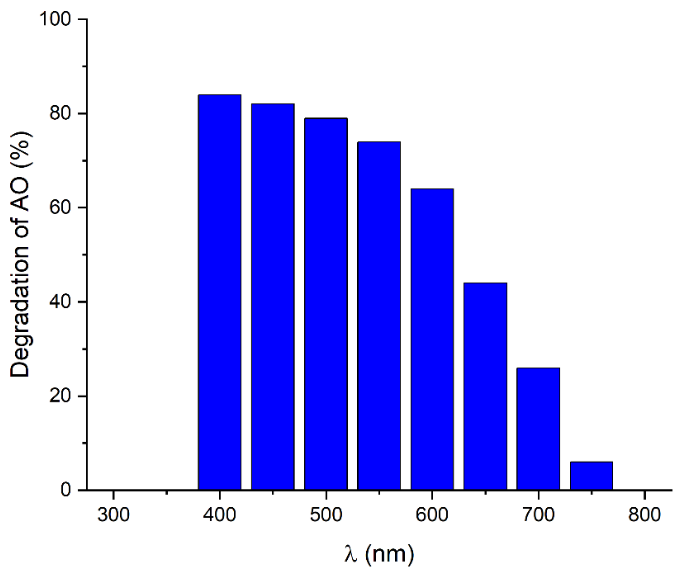

Figure S24. The photocatalytic activities of **2** at different wavelengths for the degradation of AO.

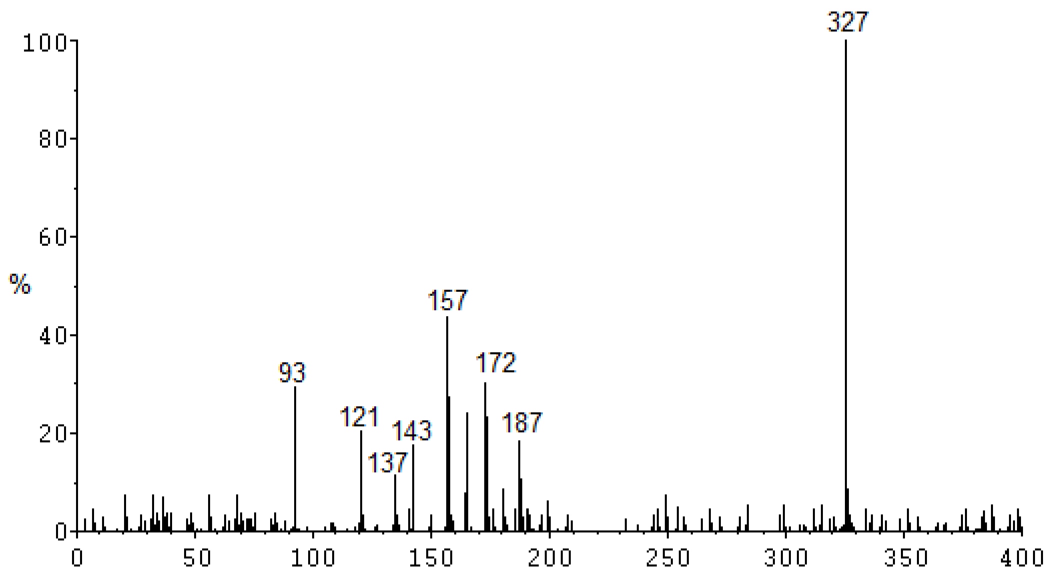

Figure S25. The ESI-MS spectrum (negative ion mode) of the reaction mixture AO by **2** after 30 min of visible-light irradiation.

Table S1. Degraded products from the photodegradation of AO identified by ESI-MS.

Products	m/z measured	Relative abundance (%)	Proposed structure
P1	327.34	100	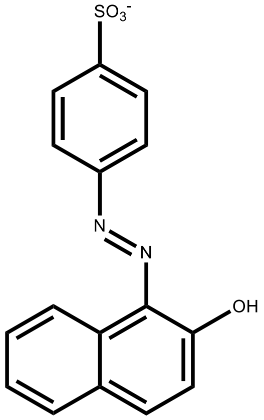
P2	187.20	18	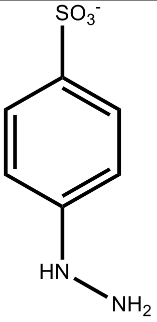
P3	172.18	30	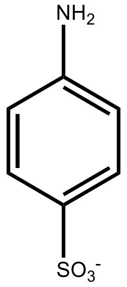
P4	157.17	43	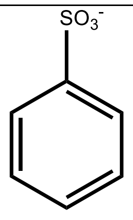
P5	143.16	19	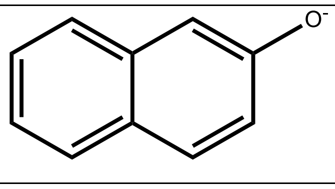

P6	137.11	17	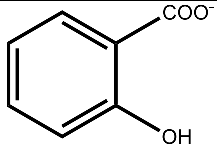
P7	121.11	20	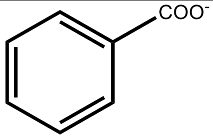
P8	93.10	29	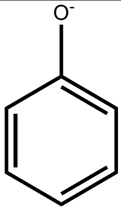
